# Influence of Laser-Based Powder Bed Fusion of Metals Process Parameters on the Formation of Defects in Al-Zn-Mg-Cu Alloy Using Path Analysis

**DOI:** 10.3390/mi15091121

**Published:** 2024-08-31

**Authors:** Biao Huang, Hongqun Tang, Jincheng Huang, Yuanxiang Jia, Liuhui Liao, Shuhuan Pang, Xu Zheng, Zhendong Chen

**Affiliations:** 1 State Key Laboratory of Featured Metal Materials and Life-Cycle Safety for Composite Structures, Guangxi University, Nanning 530004, China; 2 Key Laboratory of High Performance Structural Materials and Thermo-Surface Processing (Guangxi University), Education Department of Guangxi Zhuang Autonomous Region, School of Resources, Environment and Materials, Guangxi University, Nanning 530004, China; 3 ALG Aluminium lnc., Guangxi Key Laboratory of Materials and Processes of Aluminum Alloys, Nanning 530031, China; 4 Guangxi Yongnian Industrial Technology Research Institute of Additive Manufacturing, Nanning 530201, China

**Keywords:** laser powder bed fusion (PBF-LB/M), 7050 aluminium alloy, defects, temperature field, path analysis

## Abstract

High-strength aluminium alloys are prone to porosity and cracking during laser-based powder bed fusion of metals (PBF-LB/M) due to the complex solidification behaviour, thus limiting the preparation of high-quality aluminium alloys. In order to effectively reduce the defect formation, this study investigated the influence mechanism of different process parameters on the formation of porosity and cracks in Al-Zn-Mg-Cu alloys in the PBF-LB/M process by combining experimental and numerical simulation. The degree of influence of the process parameters on the temperature field and the temperature field on the defect formation was also quantified using path analysis. The results show that modulation of the process parameters can effectively reduce the formation of cracks and pores, although it is difficult to eliminate them. The melt pool temperature has a significant effect on the formation of porosity, and the temperature gradient has a significant effect on the formation of cracks. The degree of influence of laser power on the melt pool temperature and temperature gradient was greater than that of scanning speed, with values of 0.980 and 0.989, respectively. Therefore, the priority of modulating the laser power is higher than that of scanning speed in order to reduce the formation of defects more effectively.

## 1. Introduction

Additive manufacturing has attracted a lot of attention from both the scientific and industrial communities due to its ability to manufacture complex structures and its environmentally friendly advantages [1]. PBF-LB/M technology is a type of additive manufacturing technology [2]. It involves 3D modelling through software and using a computer to scan metal powder using a laser beam based on the built model; the metal powder is completely melted by the heat of the laser beam and eventually cooled and solidified to form the part [3,4,5]. Due to the high thermal conductivity and high laser reflectivity of aluminium alloys [6], porosity and crack defects are prone to form during the process of forming aluminium alloys with PBF-LB/M. The presence of these defects can weaken the mechanical properties of the moulded parts during their use [7,8,9].

At present, the PBF-LB/M forming of high-strength aluminium alloys remains challenging due to cracks and porosity [10]. The relationship between process parameters and defects in high-strength aluminium alloys has been studied experimentally by researchers. Tan [11] and Pekok et al. [12] investigated the evolution of densification behaviour and defect formation in 2024 aluminium alloys with different machining parameters and found that optimising the machining parameters reduces the formation of pores, whereas crack elimination is difficult to achieve due to the columnar crystals characteristic of PBF-LB/M forming. Nie et al. [13] investigated the effect of processing parameters on the formation of Al-Cu-Mg alloy specimens and found that PBF-LB/M can be optimised by careful choice of process parameters to prepare crack-free dense high-strength Al-Cu-Mg samples. Li et al. [14] investigated the effect of scanning spacing on high-strength aluminium-lithium alloys and found that too large a scanning spacing reduces the overlap between adjacent melt pools and melt paths, which can lead to incomplete powder melting and the formation of irregular pores at the gaps. Li et al. [15] investigated the effects of laser power, scanning speed, scanning distance and scanning rotation angle on the cracking behaviour of printed Al-Zn-Mg-Cu alloys and found that crack elimination was difficult to achieve through process optimisation. Oko et al. [16] found that printing 7075 using high energy densities resulted in higher part densification. Kaufman et al. [17] prepared aluminium alloy EN AW 7075 using various processing parameters and showed that high relative densities can be achieved under high laser power conditions. Tan et al. [18] found that aluminium alloys fabricated at low energy densities can have high porosity.

The process parameters affect the quality of the formed parts by controlling the temperature during the forming process, whereas non-uniformity of heating and cooling during the forming process is the root cause of the defects formation in the PBF-LB/M forming process [19]. The temperature of the PBF-LB/M forming process is difficult to measure due to its closed nature and high cooling rate. Numerical simulation is an effective method to study heat transfer in the PBF-LB/M process. The mechanism of defect formation in the PBF-LB/M process has been explored by researchers using numerical simulation [20,21,22]. Most of the current numerical simulations on PBF-LB/Med aluminium alloys focus on AlSi10Mg alloys. Li et al. [22] simulated the temperature field of AlSi10Mg powders during PBF-LB/M fabrication and explored the relationship between processing parameters and pore formation of PBF-LB/M-processed AlSi10Mg. Wei et al. [23] developed a three-dimensional numerical model to investigate the effects of laser scanning speed, laser power and hatch spacing on the thermodynamic behaviour of the molten pool during PBF-LB/M of AlSi10Mg powders and found that shallow molten pool conductivity in the presence of insufficient energy density can lead to poor adhesion between the track and the substrate and reduce the densification level. Khan et al. [24] conducted a numerical study on ANSYS 2022 and found that circular holes showed greater roundness and fewer powder chips at a laser power of 65 W and a scanning speed of 1000 mm/s. Zhou et al. [25] investigated the effect of remelting number on the quality of AlSi10Mg by analysing the melt pool changes with different remelting numbers through numerical simulations.

At present, researchers are able to study the influence mechanism of parameters on defects through numerical simulation, but to date, to be best of our knowledge, no one has attempted to quantify the influence of different parameters and simulation results on defects, so that people can clearly understand the degree of influence of different parameters and simulation results on defects, and better guide production. Path analysis is a method to study the relationship between multiple variables, solving the deficiency of traditional regression analysis, which can only analyse a single variable [26]. Li et al. [27] explored the mechanism of influence of different monomeric units of proanthocyanidins on bioactivity using a path analysis model. Azhand et al. [28] analysed the factors affecting the yield of Dragon’s Head vulgaris using path analysis. Parsamanesh et al. [29] quantified the relationship between the influence of many plant characteristics on soil activity using path analysis to obtain the most important factors affecting soil activity. These studies have utilised path analysis to examine the relationship between their variables. Since there is more than one PBF-LB/M process parameter, the process parameter influences the temperature field result, the temperature field result influences defect formation, and the relationship between the variables is complicated. Using the path analysis method can lead to an intuitive understanding of the causal relationship between these variables and the degree of influence between variables, and better guide the optimisation of process parameters.

One example of Al-Zn-Mg-Cu alloys is 7050 aluminium alloy. This alloy has the advantages of high strength, light weight and high fracture toughness [30] and has many applications in the aerospace field. With the development of aerospace technology, there is an urgent demand for 7050 aluminium alloy parts with complex shapes, and the use of PBF-LB/M technology to form 7050 aluminium alloy can well solve this problem. It should be noted that 7050 aluminium alloy has a higher Zn content than 7075 aluminium alloy, which has already been studied [31]. On the one hand, a higher Zn content can lead to the formation of more MgZn_2_ phases to improve the strength of the allow [32]; on the other hand, a higher Zn content can cause a wider solidification zone, leading to higher hot cracking sensitivity and increasing the difficulty of forming [33]. Therefore, in this paper, to address the problem of the low forming quality of 7050, on the basis of an analysis of the formation mechanism of defects through a combination of numerical simulation and experiments, based on path analysis, we propose a method to quantify the relationship between the influence of process parameters, numerical simulation, and defects, which can help to improve the forming quality of 7050 aluminium alloy and optimise the process parameters.

## 2. Experiments and Numerical Simulations

### 2.1. Powder Feedstock

**Figure 1:**
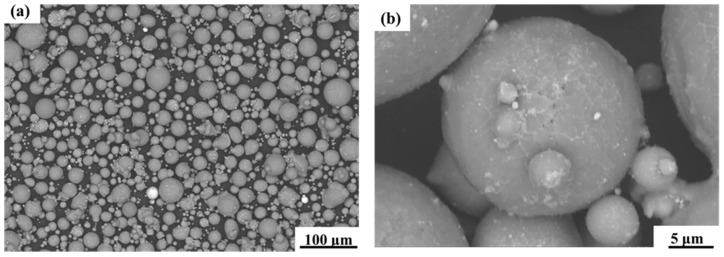
(**a**) SEM morphology of 7050 aluminium alloy powder at low magnification. (**b**) SEM morphology of 7050 aluminium alloy powder at high magnification.

The gas-atomised 7050 aluminium alloy raw material powder was supplied by Jiangsu Yongnian Laser Forming Technology Company. A fluorescence spectrum analyser (XRF, S8 TIGER from BRUKER, Karlsruhe, Germany) was used to analyse the powder’s elemental content, and the chemical composition of the powder is shown in Table 1. The powder used was observed using scanning electron microscopy (SEM, TM4000 from HITACHI, Tokyo, Japan), and the results are shown in Figure 1, where it can be found that the powder particles are close to spherical, and there are satellite spheres attached to the surface of the powder.

In order to assess the shape of the powder ground more clearly, the form factor (*FF*) of the powder was evaluated, which is defined as follows [34]:

(1) $$FF=\frac{4A\pi }{P^{2}}$$

where *A* is the area enclosed by the boundary of the particle and *P* is the perimeter of the particle outline. *FF* = 1 corresponds to a perfect circle. Image J 1.5 software was used to process the image in Figure 1a. Thresholding was adjusted for each image until the particle area appeared all black and the background appeared all white. The area and perimeter of all intact powders in the image were then quantified. The form factor of each powder was then obtained according to the formula. The results are shown in Table 2. The form factor of most of the powder is between 0.8 and 1, indicating that the particles of the powder were close to spherical and had good flowability.

**Figure 2:**
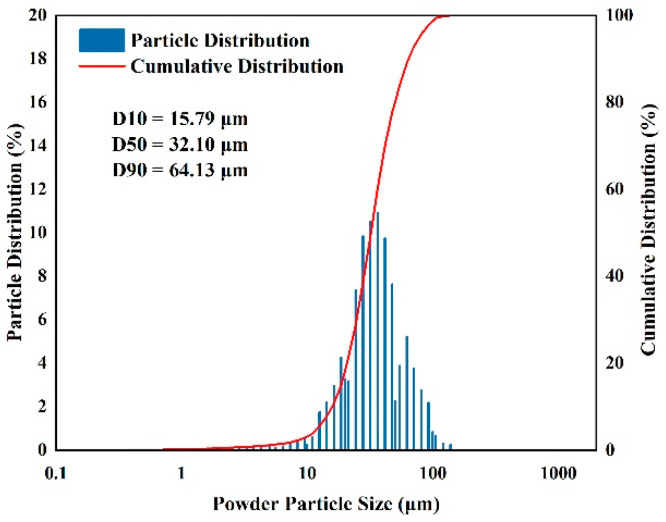
Particle size distribution of 7050 aluminium alloy powder.

The experimental powder was tested using a laser particle size meter (JL-1197 from Chengdu Jingxin Powder Test Equipment Co., Chengdu, China) and the measured sizes of the powders, with a distribution ranging from 15.79 μm (D10) to 64.13 μm (D90), are shown in Figure 2. The results of the powder morphology and particle size tests show that the powder is not prone to agglomeration, has good flowability and is suitable for PBF-LB/M printing.

### 2.2. Sample Preparation

**Figure 3:**
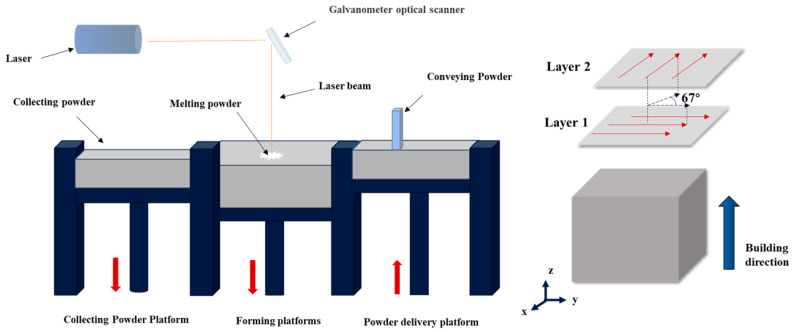
Schematic diagram of the forming principle of the experimental metal powder PBF-LB/M forming machine.

The experimental equipment used in this study was a laser printer (YLM-150 from Jiangsu Yongnian Laser Forming Technology Co., Ltd., Suqian, China). The machine is equipped with a fibre laser with a maximum output power of 500 W. The whole PBF-LB/M process was carried out in a flowing nitrogen environment with the oxygen content controlled below 0.1 vol%. The scanning direction rotation between successive layers was 67°. Cubic samples of 10 × 10 × 10 mm^3^ were fabricated. The energy density *E* is calculated using Equation (2) [35]:

(2) $$E=\frac{P}{VLh}$$

where *P* is the laser power, *h* represents the scanning pitch, *V* represents the scanning speed and *L* is the thickness of each layer. Other process parameters are shown in Table 3. The PBF-LB/M process is shown in Figure 3.

### 2.3. Microstructural Characterisation

The prepared samples were mechanically ground and polished along the longitudinal plane to obtain a proper surface finish. After processing the samples, five micrographs of different areas of the cross-section were taken using an optical microscope (OM, BH200MR from SOPTOP, Ningbo, China). Each micrograph was then processed to count the defect rate, porosity, crack rate and crack length using ImageJ. A scanning electron microscope (SEM, TM4000 from HITACHI, Tokyo, Japan) was used to characterise the morphology of the 7050 aluminium alloy powder and the microstructure and defect morphology in the PBF-LB/Med 7050 aluminium alloy. An SEM (Sigma 300 from Carl Zeiss AG, Oberkochen, Germany) equipped with electron backscatter diffraction (EBSD) was used to observe the grain morphology and stresses in the PBF-LB/Med 7050 aluminium alloy samples.

### 2.4. Numerical Simulation Process

#### 2.4.1. Finite Element Modelling

**Figure 4:**
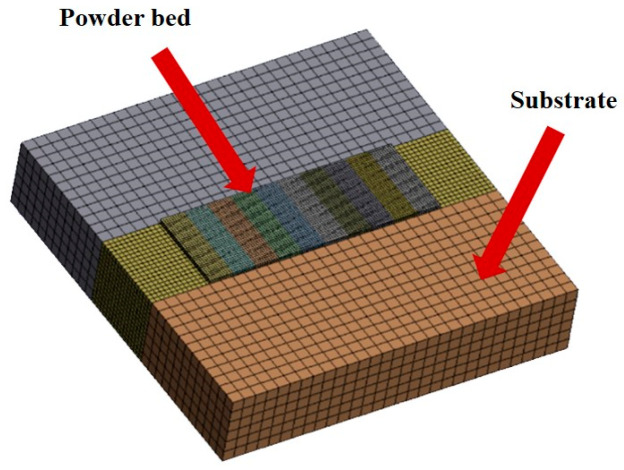
Constructed 3D finite element model.

The finite element model developed for the simulation of the PBF-LB/M process is shown in Figure 4. The size of the substrate is 1.5 × 1.5 × 0.3 mm^3^ and its mesh size is 0.05 × 0.05 × 0.05 mm^3^. The size of the powder layer is 1 × 0.35 × 0.02 mm^3^ and its mesh size is 0.01 × 0.01 × 0.01 mm^3^. The substrate under the powder layer was encrypted with a mesh size of 0.02 × 0.02 × 0.02 mm^3^. The 3D simulation model was meshed into 31570 hexahedral cells.

Due to the complex physical and chemical changes in the actual PBF-LB/M process, the finite element model is currently simplified as follows:

(1) It assumes that the entire 7050 aluminium alloy powder layer is a continuous and homogeneous medium.

(2) Changes in melting and solidification of the metal powder during laser scanning are neglected.

(3) The effect of melt pool flow on the temperature field is neglected.

(4) The absorption rate, convective heat transfer coefficient and thermal radiation of the powder are set as a fixed constant.

#### 2.4.2. Boundary and Initial Conditions

Spatio-temporal distribution of the temperature field during PBF-LB/M satisfies the differential equation for three-dimensional heat transfer [36]:

(3) $$\frac{\partial }{\partial x}(k_{x}\frac{\partial T}{\partial x})+\frac{\partial }{\partial y}(k_{y}\frac{\partial T}{\partial y})+\frac{\partial }{\partial z}(k_{z}\frac{\partial T}{\partial z})+Q=\rho c\frac{\partial T}{\partial t}$$

In the equation, *ρ* and c are the density and specific heat capacity of the material; *k_x_*, *k_y_* and *k_z_* are the thermal conductivity in the *x*, *y* and *z* directions, respectively; *T* and *t* are the temperature and time, respectively; and *Q* is the amount of heat generated, which is the amount of heat per unit volume obtained by laser radiation.

The temperature of the model at the initial moment (t = 0 s) can be expressed by the following equation [37]:

T(x, y, z)_t=0_ = T_0_ (4)

T_0_ is the initial temperature, which is 25 °C.

The boundary conditions including convection and radiation are considered in the model as follows [38]:

q_c_ = h_c_(T_0_ − T_1_) (5)

q_r_ = *ωφ*(T_0_^4^ − T_1_^4^) (6)

In the equations, q_c_ and q_r_ denote the convective and radiative heat fluxes; *ω* is the radiation coefficient; *φ* is the Stefan–Boltzmann constant (5.67 × 10^−8^ W/(m^2^·K^4^)); h_c_ is the convection coefficient; and T_1_ is the material temperature.

#### 2.4.3. Heat Source Modelling

The heat flow density generated by the laser shows a normal Gaussian distribution, and in this paper, a moving Gaussian heat source model is used as the moving heat source.

The Gaussian heat source model is expressed as follows [39]:

(7) $$Q=\frac{2kp}{\pi r^{2}}\exp[-2\frac{R^{2}}{r^{2}}]$$

*Q* is the heat flow density of the Gaussian surface heat source model in J/(m^2^·s); *r* is the radius of the laser spot in m; *R* is the distance from any point to the centre of the heat source in m; and *k* is the laser absorption rate.

#### 2.4.4. Material Parameters

The thermophysical parameters of 7050 aluminium alloy in the numerical simulation are shown in Table 4.

### 2.5. Path Analysis

In the path analysis, the laser power and scanning speed are the independent variables of the maximum temperature, cooling speed and temperature gradient in the temperature field, the maximum temperature and cooling speed are the independent variables of porosity and the temperature gradient is the independent variable of crack rate. Based on the above independent and dependent variables, first, each variable was subjected to linear regression analysis, and then, the obtained regression analyses were integrated to establish a path analysis based on the causal relationship of the respective variables. The linear regression analyses were performed using SPSS 27 software.

## 3. Results and Discussion

### 3.1. Experimental Results

**Figure 5:**
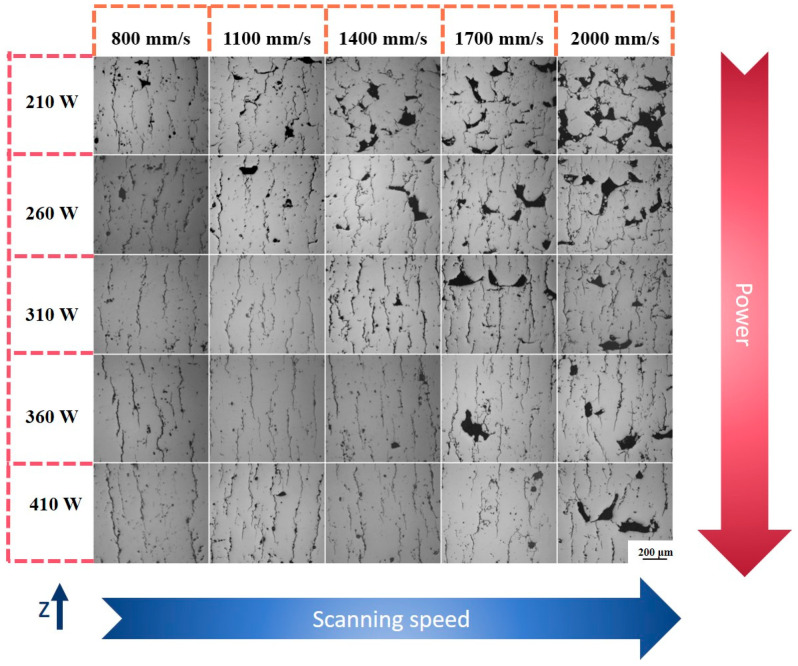
OM images of samples with different process parameters.

Figure 5 shows metallographic photographs of PBF-LB/Med 7050 aluminium alloy samples treated with different laser powers and scanning speeds. Cracks and pores can be clearly seen in all the samples, and the number of cracks and pores in the printed samples varied with the PBF-LB/M laser power and scanning speed. The shape of the cracks in the samples did not change with the change in laser power and scanning speed, and the cracks were long and thin and their directions were almost parallel to the forming direction. Furthermore, changing the process parameters could not eliminate the cracks and pores. Based on the size and morphology of the observed pores, they can be classified into three basic types: small spherical pores, small irregular pores and large pores. For the PBF-LB/Med 7050 aluminium alloy samples observed, small spherical pores were present for almost all process parameter values, large irregular pores were mainly concentrated in the samples with low power and high scanning speeds and small irregular pores, conversely, were present in the samples with high power and low scanning speeds.

**Figure 6:**
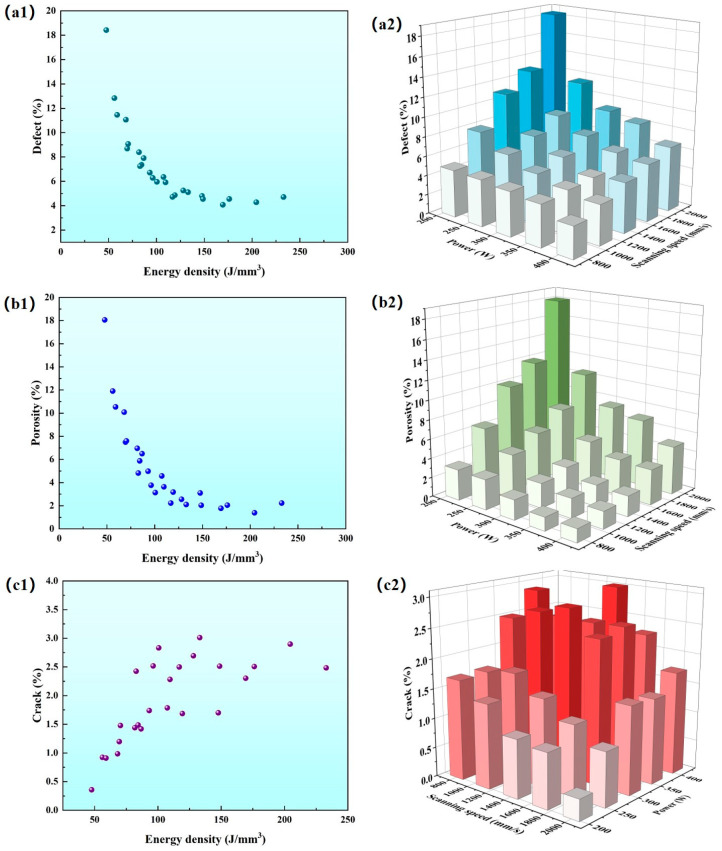
(**a1**) Scatter plot of energy density vs. defect rate; (**a2**) 3D histogram of laser power vs. scanning speed and defect rate. (**b1**) Scatter plot of energy density vs. porosity; (**b2**) 3D histogram of laser power vs. scanning speed and porosity. (**c1**) Scatter plot of energy density versus crack rate; (**c2**) 3D histogram of laser power versus scanning speed and crack rate.

The thresholding of the image was adjusted using Image J software until the defective area appeared to be all black and the background appeared to be all white. Then, the percentage of black area in the image was determined to obtain the defect rate. Next, the cracks in the image were eliminated using Photoshop 13.1 software to obtain pore-only images. Then, the percentage of black areas in the pore-only images was determined to obtain the porosity. The crack rate was obtained by subtracting the porosity from the defect rate. As shown in Figure 6, it can be found that the trends in defect rate and porosity are largely the same, decreasing with increasing laser energy density, while that of cracks is the opposite. Low defect rate and low porosity are concentrated in the high power and low scanning speed region, while low crack rate is concentrated in the region of low power and high scanning speed. Overall, the defects in the specimens are mainly affected by porosity, while cracks have little effect on the overall defects. Even if a sample with a low defect rate can be produced by optimising the parameters, it is difficult to avoid the formation of a high crack density; whereas, high porosity is difficult to avoid if a sample with a low crack density is desired. Therefore, it is difficult to achieve a 7050 alloy with low porosity and no cracks by optimising the PBF-LB/M process parameters alone.

**Figure 7:**
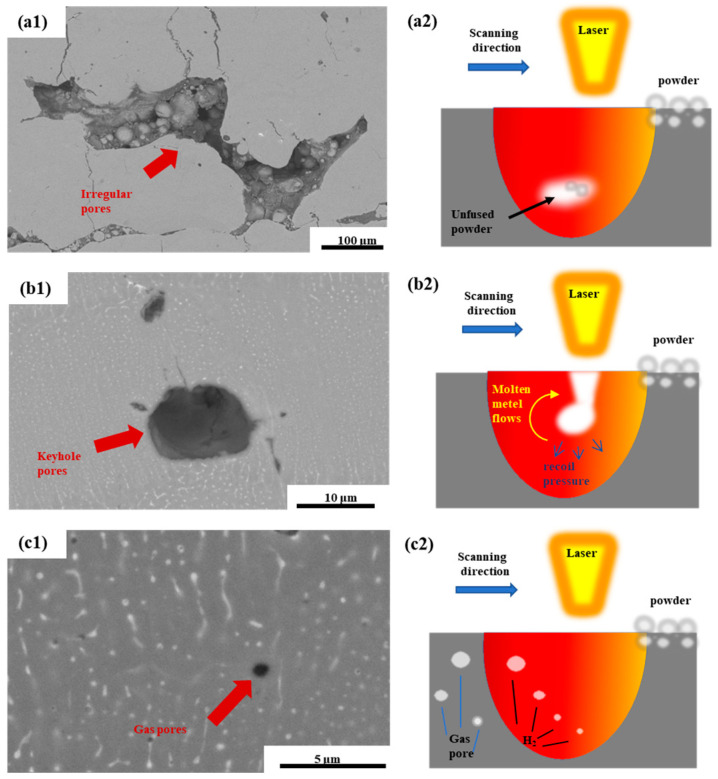
(**a1**) SEM image of irregular large pores; (**a2**) schematic diagram of the formation of irregular large pores during the printing process. (**b1**) SEM image of irregular small pores; (**b2**) schematic diagram of the formation of irregular small pores during the printing process. (**c1**) SEM image of a rounded small pore; (**c2**) schematic diagram of the formation of a rounded small pore during the printing process.

Figure 7(a1) shows an SEM image of an example of an irregular macropore. Such large irregular pores mainly exist in the case of low power and high scanning speed (low energy density). Through the observation of its microstructure, it was also found that there is some unfused spherical powder inside the pores. So, it can be shown that these irregular macropores are caused by a failure to achieve the complete fusion of the sample powder at low energy, resulting in unfused pores. As shown in Figure 7(a2), the laser energy input was too low, and only part of the powder was completely melted during the PBF-LB/M and forming process. Some particles that were not completely melted were buried underneath the powder layer leading to the creation of the observed pores [42]. The size of these unmelted pores can reach hundreds of microns and microcracks are present at their edges.

Figure 7(b1) shows the SEM picture of an example of a small irregular pore. Such small irregular pores are mainly created in the case of high power with high scanning speed (high energy density). Observation of their microstructure shows that there is no unfused powder in these pores, and these defects are related to the Al-vapour-filled depressions induced by high VED. As shown in Figure 7(b2), such pores are mainly formed by the collapse of the pore wall and the generation of metal vapour. This is due to the high laser energy irradiation to the powder bed, which increases the depth of the molten pool of the powder bed. The high laser energy also forms a high temperature to make the surface of the molten pool locally vaporise to form a cavity, and the molten pool then forms a depression due to the recoil pressure. The vortex flow results in the rear wall being very unstable, making it prone to move towards the front wall and collapse, eventually forming irregular pores [43,44]. Small pores of this type in 7050 aluminium alloys exist either independently as pores or with cracks around the edges as in the case of large pores.

Figure 7(c1) shows an example of a small spherical pore, which can be found in the samples with all process parameters shown in Figure 5. These spherical defects are usually defined as air pores. As the powder is in contact with air, the water vapour in the air adheres to the powder, as shown in Figure 7(c2), and during the PBF-LB/M process, a reaction occurs, namely, 2Al + 3H_2_O = Al_2_O_3_ + 3H_2_, and under the high cooling rate of PBF-LB/M, the hydrogen generated fails to spill out of the molten pool during the solidification stage of the molten pool, which in turn forms these types of rounded pores in the solidified samples [45].

**Figure 8:**
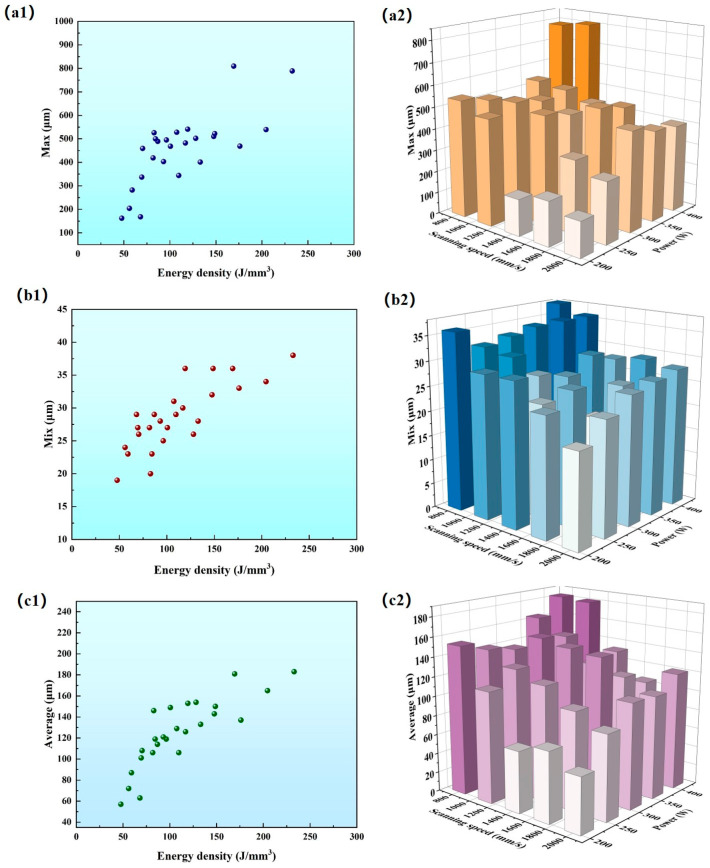
(**a1**) Scatter plot of energy density versus crack maximum length; (**a2**) 3D histogram of laser power versus scanning speed and crack maximum length. (**b1**) Scatter plot of energy density versus crack minimum length; (**b2**) 3D histogram of laser power versus scanning speed and crack minimum length. (**c1**) Scatter plot of energy density versus average crack length; (**c2**) 3D histogram of laser power versus scanning speed and average crack length.

Figure 8 illustrates the positive linear relationship between crack length and energy density. As the laser energy increases, the longest extent, the longest length and the average length of the cracks increase, with short cracks concentrated in the region of high scanning speed and low laser power, and long cracks concentrated in the region of low scanning speed and high laser power.

**Figure 9:**
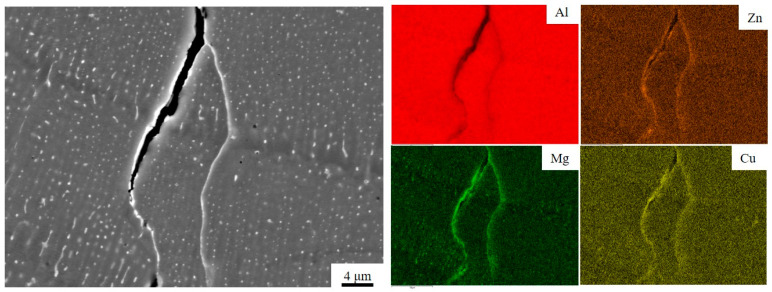
Cracked SEM images and corresponding EDS plots of PBF-LB/Med 7050 aluminium alloy.

From Figure 9, it can be found that there is both an enrichment in elements at the cracks and elemental segregation during solidification. This is consistent with the findings of Yao [46] and Tan [11], which indicate the existence of elemental segregation at grain boundaries. The presence of elemental segregation at cracks indicates that cracks tend to form and expand at grain boundaries.

**Figure 10:**
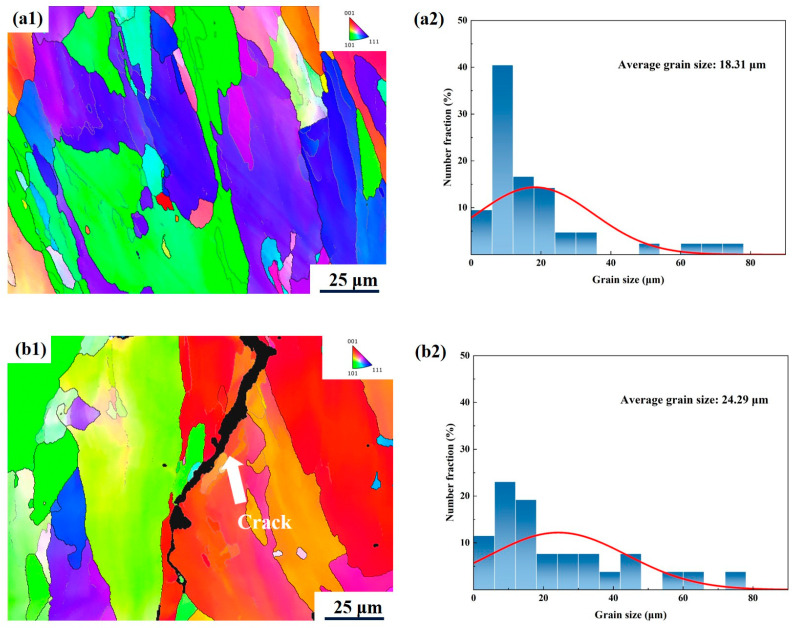
IPF map (**a1**) and grain size distribution (**a2**) for the sample with E = 47.7 J/mm^3^. IPF map (**b1**) and grain size distribution (**b2**) for the sample with E = 100.7 J/mm^3^.

EBSD was used to further analyse the microstructural characteristics of the PBF-LB/Med 7050 samples at different energy densities (47.7 J/mm^3^ and 100.67 J/mm^3^). According to Figure 10(a1,b1), it can be seen that most of the grains grow along the build direction during PBF-LB/M, and coarse columnar grains grow around the cracks. From Figure 10(a2,b2), it can be seen that the average grain size at an energy density of 100.67 J/mm^3^ is 24.29 μm, and the maximum grain size is 75.9 μm, while the average grain size at an energy density of 47.7 J/mm^3^ is 18.31 μm, and the maximum grain size is 73.80 μm. This indicates that with an increase in energy density, the grain size is also increased, as is the size of the columnar grains growing in the vertical direction, and the trend of the vertical growth of columnar grains is more and more obvious.

**Figure 11:**
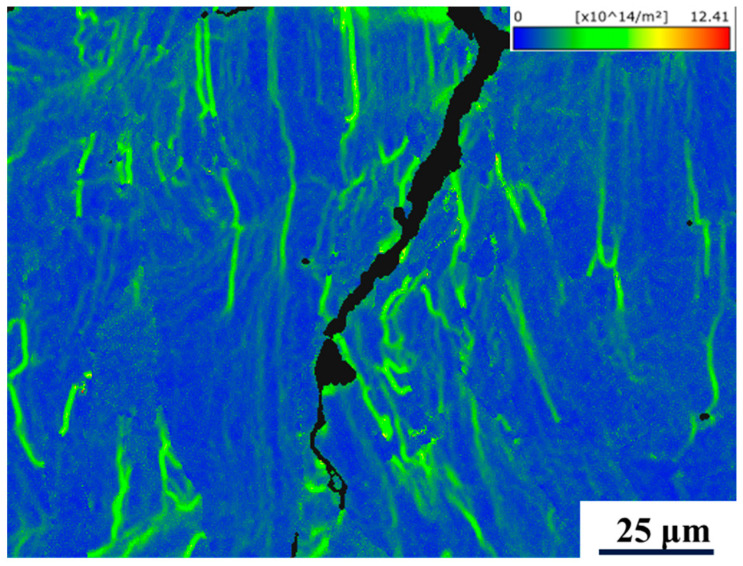
GND map for a sample with E = 100.7 J/mm^3^.

The GND map can reflect the dislocation density of the sample, and high dislocation density indicates severe grain deformation and high stress [47]. In this paper, the GND diagram is used to reflect the stress magnitude situation. As shown in Figure 11, the crack in the figure is purely surrounded by higher dislocation density, which confirms that high stress leads to crack generation and extension, and stress is the driving force for crack sprouting and extension—the higher the energy density, the higher the crack extension driving force and the longer the crack length.

### 3.2. Numerical Simulation Results

**Figure 12:**
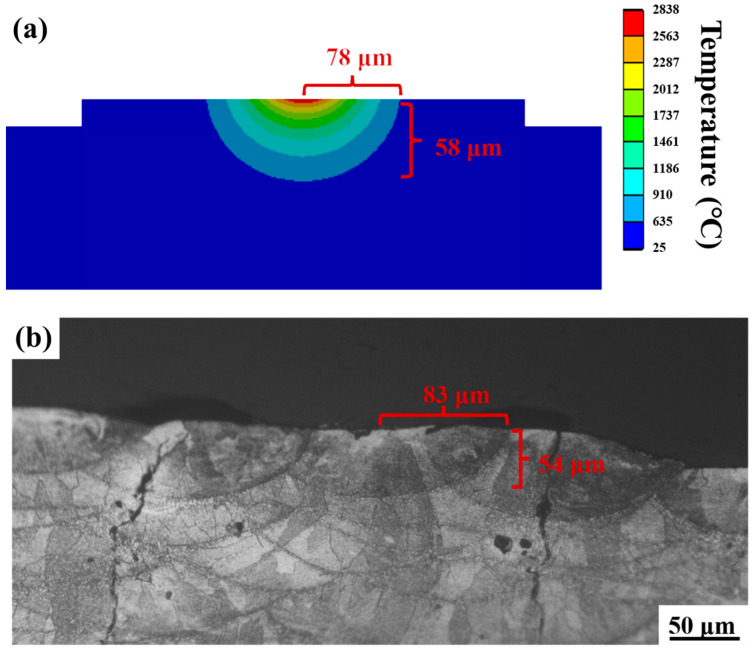
Model validation: (**a**) temperature field simulation results; (**b**) experimental OM image of the melt pool.

The prediction accuracy of the fine-scale model was verified by measuring the depth and width of the melt pool. Figure 12 illustrates the comparison between the experimental and numerical results for a melt pool with p = 410 W and v = 800 mm/s. The area above the light blue region in Figure 12a indicates the melted area and the dark blue area indicates the unmelted area. The chosen 7050 alloy has a melting point of 635 °C, so the 635 °C isotherm is the melt pool boundary. By comparing the metallographic pictures with the numerical simulation melting pool results, the error in the melting depth of the simulation is 6.0% and the error in the melting width of the simulation is 7.4%; therefore, it can be concluded that the error between the modelled melting pool and the actual melting pool is not significant, and the simulation results are in good agreement with the experimental results, so the accuracy and reliability of the developed model are reasonable for further research.

**Figure 13:**
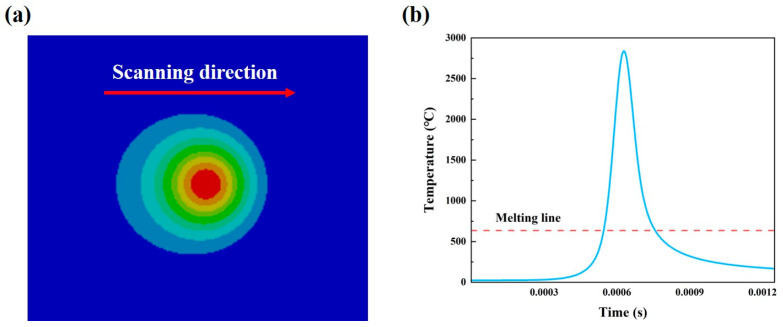
(**a**) Temperature distribution on the upper surface of the melt pool; (**b**) temperature distribution at the centre of the spot at different times.

As shown in Figure 13a, the melt pool shape on the upper surface of the specimen is elliptical and symmetrical from top to bottom, and the isotherms of the temperature field are denser at the front end of the melt pool (in the direction of the scanning speed) than the back end of the melt pool. This is mainly due to the fact that the material at the back end of the bath condenses into a solid quickly after the laser beam leaves, resulting in an increase in thermal conductivity and a faster transfer of heat to the powder bed. At this time, the material at the front end of the melt pool absorbs energy and rapidly warms up, melts into a liquid, the thermal conductivity decreases and the pool eventually forms a shape similar to an ellipse before this morphology. As shown in Figure 13b, with the laser beam scanning towards and away from each centre point, the temperature of the centre point will periodically rise suddenly and then fall sharply.

**Figure 14:**
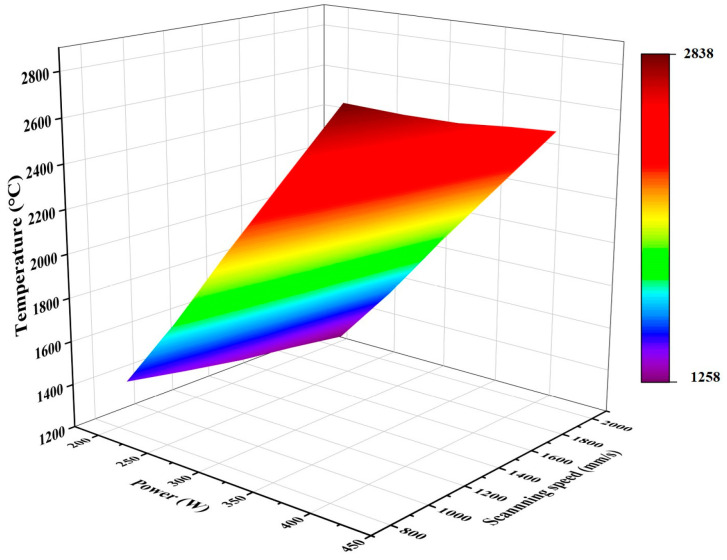
3D mapped surface plot of laser power versus scanning speed and melt pool temperature.

Figure 14 shows the maximum temperature that can be reached by the molten pool of PBF-LB/M-formed 7050 powder under different process parameters. As shown in the figure, under the condition of constant scanning speed, the laser power is positively proportional to the temperature of the molten pool. When the scanning speed is set at 800 mm/s, as the laser power increases from 210 W to 410 W, the temperature increases from 1432 °C to 2838 °C; thus, the temperature change is 1406 °C. Conversely, the temperature of the molten pool is inversely proportional to the scanning speed. When the laser power is set at 410 W, as the scanning speed is increased from 800 mm/s to 2000 mm/s, the temperature decreases from 2838 °C to 2490 °C; thus, the temperature change is 348 °C. The temperature of the molten pool is also reduced by an increase in scanning speed. It can be seen that the effect of scanning speed on the peak temperature is not as large as that of laser power. This is because the scanning speed of the selective laser melting technology is very fast; therefore, the powder is subjected to laser irradiation for only a split second, so its peak temperature is more affected by the laser power.

**Figure 15:**
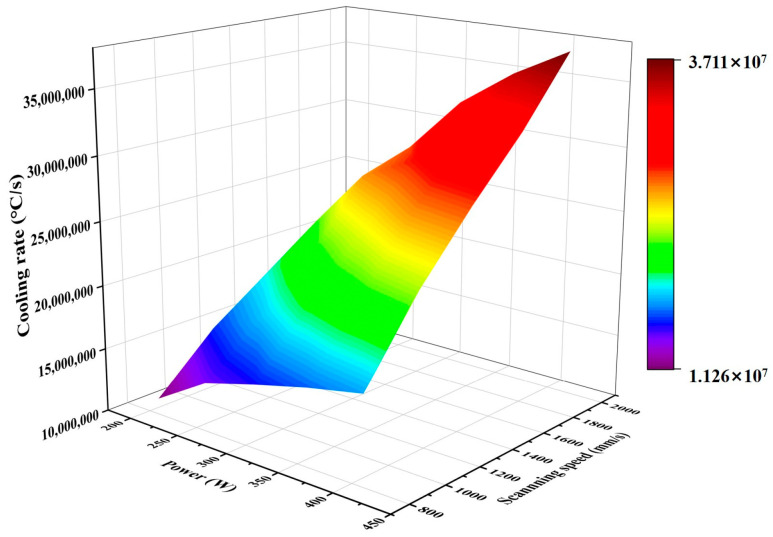
3D mapped surface plot of laser power versus scanning speed and melt pool cooling rate.

As Figure 15 shows, laser power and scanning speed have an important effect on the cooling rate, and higher laser power or a smaller scanning speed will lead to an increase in the cooling rate. The highest scanning speed is 2000 mm/s, and the laser stays in one place for the shortest time at this speed, which leads to the molten pool not having enough time to exchange heat with the surrounding environment, resulting in a high cooling rate.

**Figure 16:**
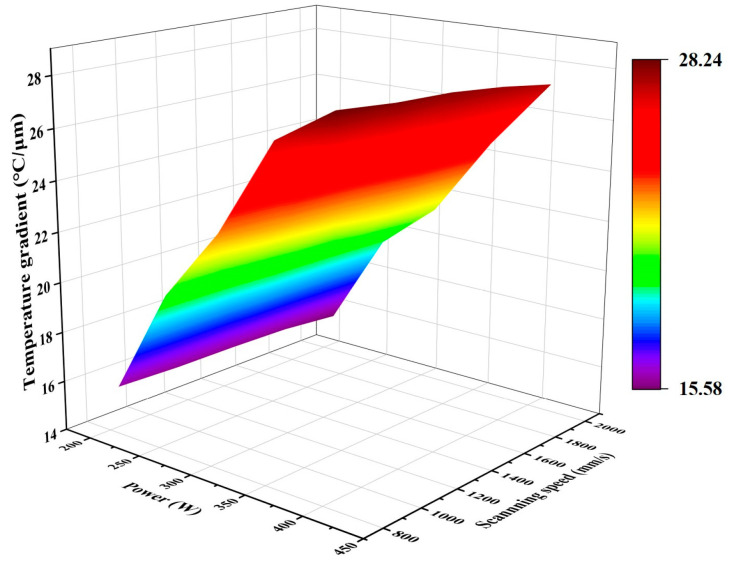
3D mapped surface plot of laser power versus scanning speed and temperature gradient.

From Figure 16, it can be seen that the laser power has a positive correlation with the temperature gradient—the higher the laser power, the larger the temperature gradient—while, the scanning speed has a negative correlation with the temperature gradient—the faster the scanning speed, the larger the temperature gradient. Similarly, the temperature gradient responds to the residual stress—the higher the temperature gradient, the higher the residual stress [48]. As the laser power increases or the scanning speed decreases, the residual stress increases; therefore, as the stress is the driving force of crack expansion, crack length will increase with an increase in laser energy density.

**Figure 17:**
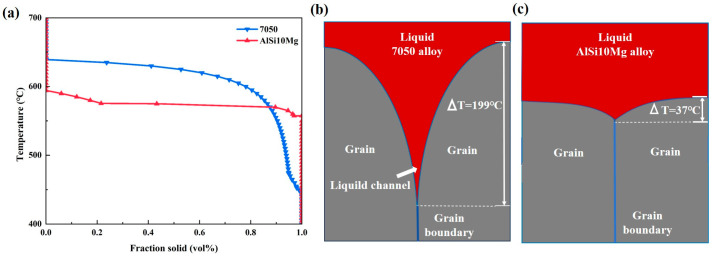
(**a**) Solidification curves of 7050 alloy and AlSi10Mg; (**b**) liquid reflux channel of 7050 aluminium alloy; (**c**) liquid reflux channel of AlSi10Mg.

Figure 17a shows the solidification curves of 7050 aluminium alloy and AlSi10Mg calculated using JMatPro 7.0 software. The results show that 7050 aluminium alloy completely solidifies from liquid to solid with a larger range of temperatures, which means that it takes more time to completely solidify. Similarly, 7050 aluminium alloy takes more time to solidify than AlSi10Mg, as AlSi10Mg has a narrower liquid reflux channel for grain growth. Figure 17b,c show the liquid reflux channel of 7050 aluminium alloy compared to that of AlSi10Mg during solidification processing. Due to the high solidification interval of 7050 aluminium alloy, the narrow channels formed in the intergranular space are more elongated than those of AlSi10Mg, which results in more time or more liquid phase to backfill the intergranular channels being required than for AlSi10Mg. Due to the short solidification time of the liquid phase during PBF-LB/M, the high solidification interval of 7050 aluminium alloy will make it difficult for the liquid to have enough time to fill the dendritic intergranular gaps in the final solidification stage, which, ultimately, leads to cracking under solidification shrinkage and tensile stress. Therefore, at the high cooling rate of PBF-LB/M, 7050 aluminium alloy is prone to cracking, whereas AlSi10Mg aluminium alloy AlSi10Mg experiences few cracks.

### 3.3. Path Analysis Results

Linear regression was used to develop a model to predict the temperature of the molten pool, cooling rate, temperature gradient and pores and cracks in the samples. The resulting prediction model equations are presented in Table 5. The validity of the obtained models was assessed by the coefficient of determination (R^2^), ANOVA test. the R^2^ values ranged from 0.499 to 0.998, which indicated that the independent variables of the obtained regression models could explain the variations of the dependent variables well. In addition, according to the ANOVA results, the *p*-values of all predictive equations were <0.001, indicating that the regression equations were significant and these equations were statistically significant (Table 5).

**Figure 18:**
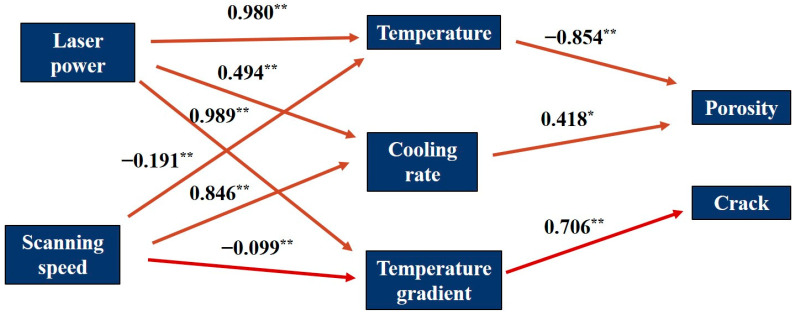
Path diagram showing the effect of process parameters on temperature field results and of temperature field results on defects. Numerical values indicate path coefficients between variables. Solid arrows indicate impact. ** and * are significant at the 0.001 and 0.01 levels of probability, respectively.

Tan et al. [11] also found a significant effect of power and scanning speed on the formation of defects in PBF-LB/Med aluminium alloys, but they did not state which process parameter had a large effect on the defects. This study quantifies the extent of the influence of process parameters on the temperature field and the temperature field on defects by developing a path analysis model that shows the significant influence of the process parameters on defect formation. The path diagram was used to determine the relationship between the influence of process parameters on the temperature field and defects, which was integrated with the results of the regression analysis (Figure 18). As can be seen, all dependent independent variables have a significant effect on the dependent variable. From the path coefficients, it can be seen that the laser power has a greater effect on the melt pool temperature and temperature gradient, which is much higher than the scanning speed, and a relatively small effect on the cooling speed, compared to the scanning speed. The temperature has a greater and negative effect on the formation of pores compared to the cooling rate, while the cooling rate has a positive effect on the pores. The temperature gradient has a significant positive effect on crack formation.

An increase in laser power gives the atoms higher kinetic energy, which allows the atoms to fill more pores in a limited time; the lower the scanning speed, the longer the laser acts on the powder, and the more energy the atoms obtain [49]. From Figure 15, it can be seen that the cooling rate of the molten pool under the process parameters of this experiment ranges from (1.126 × 10^7^–3.711 × 10^7^), and due to the characteristics of the extremely fast cooling rate under the PBF-LB/M process, this makes the change in the energy gained under different scanning speeds not as large as the change in the laser power that directly changes the laser power, which therefore results in the laser power having a greater effect on the porosity compared to the scanning speed. Similarly, due to the extremely fast cooling rate characteristic of PBF-LB/M, it is difficult to achieve rapid temperature conduction in very short time changes, resulting in a greater degree of influence of power on the temperature gradient than scanning speed. Therefore, the mitigation of defects by optimising laser power has a higher priority than controlling scanning speed.

## 4. Conclusions

Based on the above study of defects in PBF-LB/Med-formed 7050 aluminium alloy, the main conclusions that can be drawn can be summarised as follows:

(1) In the process of PBF-LB/M forming 7050 aluminium alloy, it was found that the main types of defects consist of porosity and cracks. Among them, porosity is mainly classified into unfused porosity formed at low energy and lock pores formed at high energy density, while air pores are a common defect that exists at all parameter settings. Cracks mainly appear as vertical cracks along the printing direction. These cracks are difficult to eliminate by optimising process parameters and become longer with increasing energy density.

(2) In the PBF-LB/Med 7050 aluminium alloy, cracks form mainly due to the high solidification range and thermal stresses caused by the temperature gradients. Due to the formation of long intergranular channels due to the wide solidification range and the higher cooling rate (10^7^ °C/s) during PBF-LB/M, the liquid metal is not able to adequately fill the elongated voids formed by the longer crystalline channels. Together with the thermal stress caused by the temperature gradient, cracks are eventually formed by expansion during the solidification stage.

(3) The effect of laser power on the melt pool temperature and temperature gradient is greater than that of scanning speed, with levels of 0.980 and 0.989, respectively. The melt pool temperature and temperature gradient play a major role in the formation of porosity and cracks, with effect levels of 0.854 and 0.706, respectively. Therefore, the laser power moderates the formation of porosity and cracks more than the scanning speed. In the process of regulating the laser power and scanning speed to reduce the defects, the laser power should be regulated preferentially to reduce the number of defects more effectively.

## Figures and Tables

**Table 1:** Chemical composition of 7050 aluminium alloy powder (wt.%).

Alloy	Al	Zn	Mg	Cu	Zr	Si	Fe	Ti
7050	Bal.	5.87	1.21	1.98	0.084	0.033	0.023	0.022

**Table 2:** Form factor of 7050 aluminium alloy powder.

Form Factor (Sphericity)	0–0.4	0.4–0.5	0.5–0.6	0.6–0.7	0.7–0.8	0.8–0.9	0.9–1
Number fraction (%)	0	1	2	9	15	24	49

**Table 3:** PBF-LB/Med 7050 Specimen Parameter Table.

Process Parameters	Numerical Value	Unit
Laser power (P)	210, 260, 310, 360, 410	W
Scan speed (V)	800, 1100, 1400, 1700, 2000	mm/s
Hatch space (h)	110	μm
Layer thickness (L)	20	μm

**Table 4:** Thermophysical physical parameters of 7050 aluminium alloy used in the simulation [18,40,41].

Properties	Values
Density (kg/m^3^)	2830
Specific heat J/(kg·K)	860
Conductivity W/(m·K)	157
Convective heat transfer coefficient W/(m^2^·°C)	80
Laser absorption rate	0.15

**Table 5:** Predictive model equations and R^2^ and *p*-values for temperature, cooling rate, temperature gradient, porosity and cracking.

Prediction Model Equation	R^2^	*p*-Value
Temperature = 246.433 + 6.61 P − 0.215 V	0.998	<0.001
Cooling rate = −10,576,413.1 + 47,602.848 P + 13,586.573 V	0.960	<0.001
Temperature gradient = 5.214 + 0.059 P − 0.001 V	0.987	<0.001
Porosity = 13.672 − 0.007 Temperature + 2.403 × 10^−7^ Cooling rate	0.679	<0.001
Crack = −0.702 + 0.117 Temperature gradient	0.499	<0.001

## Data Availability

The data presented in this study are available on request from the corresponding author.
